# Challenges and Potential in Increasing Lutein Content in Microalgae

**DOI:** 10.3390/microorganisms9051068

**Published:** 2021-05-15

**Authors:** Yuxiao Xie, Xiaochao Xiong, Shulin Chen

**Affiliations:** Department of Biological Systems Engineering, Washington State University, Pullman, WA 99164-6120, USA; yuxiao.xie@wsu.edu (Y.X.); xcxiong@wsu.edu (X.X.)

**Keywords:** lutein, storage capacity, lipid droplets

## Abstract

Research on enhancing lutein content in microalgae has made significant progress in recent years. However, strategies are needed to address the possible limitations of microalgae as practical lutein producers. The capacity of lutein sequestration may determine the upper limit of cellular lutein content. The preliminary estimation presented in this work suggests that the lutein sequestration capacity of the light-harvesting complex (LHC) of microalgae is most likely below 2% on the basis of dry cell weight (DCW). Due to its nature as a structural pigment, higher lutein content might interfere with the LHC in fulfilling photosynthetic functions. Storing lutein in a lipophilic environment is a mechanism for achieving high lutein content but several critical barriers must be overcome such as lutein degradation and access to lipid droplet to be stored through esterification. Understanding the mechanisms underlying lipid droplet biogenesis in chloroplasts, as well as carotenoid trafficking through chloroplast membranes and carotenoid esterification, may provide insight for new approaches to achieve high lutein contents in algae. In the meantime, building the machinery for esterification and sequestration of lutein and other hydroxyl-carotenoids in model microorganisms, such as yeast, with synthetic biology technology provides a promising option.

## 1. Introduction

Lutein ((3R, 3′R, 6′R)-β, ε-carotene-3, 3′-diol) is a C40 hydroxyl-carotenoid with a chemical structure that has an unsaturated polyene chain as the skeleton, one ε-ring and one β-ring on each side of the skeleton, with a hydroxyl-group on the 3′ site of each ring. Lutein is primarily synthesized by plants and algae with two forms. In the first configuration, lutein is present as a “structural form”, and its function is primarily involved in light-harvesting and nonphotochemical quenching [1,2]. In the second form, with one or two fatty acids bound to the hydroxyl-groups, lutein is converted into “oil form” that is accumulated in lipid vessels of many flowers and fruits [3].

Lutein has demonstrated use as an antioxidant, a food coloring agent and a nutraceutical, and is thus widely used in cosmetics, food additives and drugs [4,5]. The conjugated double bonds of lutein provide substantial antioxidant property. Because the human body cannot synthesize lutein and other carotenoids, dietary intake is vital to human health. Lutein and its isomer zeaxanthin provide unique health benefits to the human macular region. These are the only two carotenoids that are greatly enriched in the human retinal macula region to improve visual function, filter blue light and prevent oxidative damage [6]. In fact, a six milligram of daily intake of lutein can significantly lower the risk of age-related macular degeneration (AMD) [7,8]. Studies also suggest that lutein may reduce the risk of some cancers and prevent heart disease and stroke [9,10,11,12]. These beneficial effects of lutein on human health have prompted increased demand for lutein and resulted in a significant global lutein market.

Algal-based lutein production provides a promising alternative to that extracted from marigold, although currently the commercial supply of lutein mainly depends on farming and extracting marigold petals in China, India and Mexico [4,13,14]. Compared with marigold, microalgae have several unique characteristics. Microalgae grow much faster than marigold, leading to higher lutein annual productivity. They can be cultured in large-scale fermenters, photobioreactors or even open ponds. Therefore, microalgae-based lutein production is an industrially scalable process. It is also less susceptible to seasonal, climatic and land limitations than marigold-based lutein production [4,13,15].

In the past two decades, several microalgae species such as *Chlorella* spp., *Scenedesmus* spp., *Muriellopsis* spp., and *Dunaliella* spp. have been investigated for lutein production [16,17,18,19]. Considering the high cost of cell harvesting, cell disruption and pigment extraction in microalgae-based lutein production, the studies suggest that lutein content need be greater than 10 mg/g to be commercially feasible [13]. However, algal lutein content averages around 5 mg/g of dry cell weight (DCW). Therefore, microalgae-based lutein production has not been commercialized yet, due to low lutein content in microalgal cells.

Extensive studies have explored new algal species with naturally high lutein content, and have enhanced lutein content with different cultivation methods in the recent years (Table 1). Xie et al. isolated a lutein-rich microalgae species, *Chlorella sorokiniana* FZU60 [20]. After optimizing sodium acetate and nitrate concentration in the growth medium, maximum lutein content, lutein productivity and biomass productivity reached 9.65 mg/g, 4.86 mg/L/d, and 503.55 mg/L/d, separately. Pilot-scale cultivation of *C. sorokiniana* FZU60 in a 50 L column photobioreactor was conducted and optimized. Optimum lutein content, lutein yield and lutein productivity were achieved at 9.51 mg/g, 33.55 mg/L, and 4.67 mg/L/d, separately, under a novel, two-stage cultivation mode [21]. Heo et al. optimized and evaluated the lutein production of microalgae *Parachlorella* sp. JD-076 under different types of photobioreactors. Maximum lutein content of 11.8 mg/g, with a lutein productivity of 25.0 mg/L/d, was achieved by exposing the strain to 1000 μmol m^−2^ s^−1^ of light intensity [22].

Efforts have also been made on strain improvements for increasing lutein content. Random mutagenesis has been used as a useful technique to improve strains and isolate lutein-rich algal mutants [25]. Chemical mutagens and UV-irradiance were attempted to generate mutant strains. High lutein yield algal mutants were isolated by resistance to inhibitors of the carotenogenic pathway. In this way, several algal mutants with high lutein content have been isolated (Table 2). For example, Cordero et al. obtained several high lutein-yield *C. sorokiniana* mutants through random mutagenesis with N-methyl-N’-nitro-nitrosoguanidine (MNNG). The highest lutein content, 7.0 mg/g, was achieved by the mutant DMR5 and DMR-8 [26]. A 7.52 mg/g lutein content was achieved with the mutant strain *C. sorokiniana* MB-1-M12 under mixotrophic conditions [24]. *Chromochloris zofingiensis* (also named as *Chlorella zofingiensis*) could accumulate lutein, zeaxanthin, β-carotene and astaxanthin under different cultivation conditions [27]. After mutagenesis by MNNG, Huang et al. isolated a *C. zofingiensis* mutant with a dysfunctional *BKT1* gene. In this mutant, the content of lutein, zeaxanthin and β-carotene reached 13.81 ± 1.23, 7.00 ± 0.82, and 7.18 ± 0.72 mg/g, separately [28].

Metabolic and genetic engineering are also used as effective tools to manipulate the rate-limiting step of the metabolic pathway for increasing lutein content and productivity in algal strains [33]. Although genetic engineering of algae is still in the initial stages, the use of this technique to enhance lutein content in microalgae has made progress in recent years. For example, new molecular technologies such as CRISPR/Cas9 genome engineering technology and genome sequencing have improved understanding of lutein metabolism regulation in algae species [34,35,36,37].

Phytoene synthase (PSY) and phytoene desaturase (PDS) are essential enzymes that catalyze rate-limiting carotenoid biosynthesis steps in plants and algae. Overexpression of these genes may increase lutein content in algal cells. For example, the heterologous *PSY* gene from *D. salina* and *C. zofingiensis* has been overexpressed in *C. reinhardtii* [29,30]. The *PSY* transformants exhibited a 1.6-fold and 2.0-fold increase of lutein content in transgenic cells compared with the wild type. The endogenous *PDS* gene was overexpressed in *C. zofingiensis* and resulted in a 32.1% increase in the total carotenoid content [31].

Another approach taken is to enhance the enzymatic activity of PSY. The orange (*OR*) gene encodes a plastid-associated protein, which triggers the chloroplast to chromoplast differentiation in plants. Many studies have focused on the OR protein’s positive effect on the post-transcriptional regulation of carotenoids by stabilizing the PSY protein and maintaining the PSY protein level [38,39,40]. Recently, an *OR* gene from arabidopsis (*AtOR*) was expressed in the alga *C. reinhardtii*, resulting in a 1.9-fold and 1.7-fold increase in lutein β-carotene, respectively [32].

In sum, research on the production of microalgae-based lutein has made remarkable progress. Recent work has approached the commercialization threshold of microalgal-based lutein production. However, as a carotenoid producer, the level of lutein accumulation in microalgae species is still far below the level of β-carotene concentrates in *Dunaliella* spp. (up to 14% of DCW) and astaxanthin in *Haematococcus* spp. (up to 5% of DCW) [41,42]. Therefore, further research is warranted to elucidate the mechanisms of lutein biosynthesis and regulation and to identify potential limiting factors of lutein accumulation in microalgae.

## 2. Lutein Sequestration Versus Structural Function

Sequestration capacity is critical for lutein accumulation because the structural form of this pigment, with an esstantial role in fulfilling photosynthetic functions, only has a minimal content.

The accumulation of lutein is the ultimate result of three major processes: biosynthesis, degradation and sequestration. The biosynthesis of lutein has been extensively studied and reviewed. This includes the metabolic pathway, transcript regulation and post transcript regulation [2,43,44,45]. The degradation of lutein and other carotenoids by carotenoid cleavage dioxygenases (CCDs) has also garnered much attention [46,47]. Moreover, carotenoid sequestration has been recognized as an essential factor for carotenoid accumulation in plants [45,48,49]. In contrast, the limitation of lutein sequestration in algae has been neglected. This work thus focuses on surveying current findings and knowledge related to lutein sequestration in microalgae.

**Figure 1:**
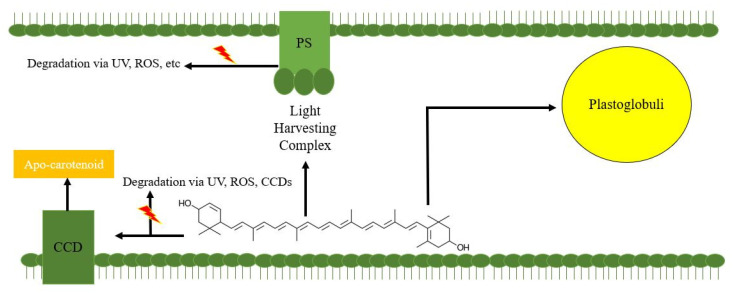
The sequestration of lutein in photosynthetic organisms. PS: photosystems. UV: ultraviolet light. ROS: reactive oxygen species. CCD: carotenoid cleavage dioxygenases.

As illustrated in Figure 1, the biosynthesis of lutein occurs on the inner chloroplast membrane of plants and algae [50]. Due to the lipophilic characteristic of the unsaturated polyene chain and the hydrophobic hydroxyl groups on the rings, it is difficult for lutein to float freely in the hydrophilic environment of the chloroplast and attaches tightly to the membrane. Thus, once lutein is synthesized, lutein accumulates on the inner chloroplast membrane. Without proper sequestration, this leads to feedback inhibition on lutein synthesis or degradation by ROS or CCDs. Therefore, lutein sequestration is essential for lutein accumulation, and the storage capacity of lutein might determine the upper limit of lutein content in the organisms.

Based on current knowledge, lutein could bond to LHC-associated proteins as a structural and functional pigment involved in light-harvesting and nonphotochemical quenching (NPQ) in plant green tissues and microalgae [1,51]. Lutein could also be stored in carotenoid-lipoprotein substructures and the plastoglobuli, an oil environment, within the chromoplasts of flower petals such as marigold. It was found that lutein could be massively accumulated in the plastoglobuli of the petals as an “oil form” [52,53,54]. In contrast, lutein content is usually low in the leaves as a “structural form” in marigold. This difference in the two lutein sequestration methods might lead to the variance in the level of lutein accumulation. Therefore, exploring the storage capacity of lutein under the two methods can provide a rough estimation of the level of content in lutein producing organisms.

A standard is required to estimate the storage capacity of lutein in LHCs and lipid droplets, but there is not a widely accepted, accurate standard to address this problem. Based on current knowledge, the mass ratio between the accumulated lutein and the lutein storage medium can possibly be used to estimate the capacity of lutein sequestration.

### 2.1. Estimation Of Lutein Storage Capacity in LHCs

As understanding of photosystems (PS) structure in plants and microalgae advances, the amount of lutein and other carotenoid associated with the PS and LHC protein complex becomes clearer [51,55,56]. Most carotenoids involved in photosynthesis are bound to the light-harvesting chlorophyll a/b-binding (Lhcb) protein. The core of photosynthetic systems and cytochrome b6f complex can also attach carotenoids. Lhcb proteins have a similar structure and conserved binding sites for 11–14 chlorophyll molecules and 2–4 carotenoid molecules through plants and microalgae. Here, the Lhcb protein is assumed as the direct lutein container for the purpose of estimating the storage capacity of lutein in structural form. Lhcb protein exhibits a 28.1 kDa mass weight and four carotenoid binding sites, L1, L2, N1, and V1. Lutein can bond to the L1 and L2 sites of Lhcb. Neoxanthin and violaxanthin can bond to the N1 and V1 sites, respectively. Thus, based on the 568.87 Da mass of lutein, lutein’s storage capacity on Lhcb proteins is estimated as 4.1%. In addition, biochemical data suggested that these bonding sites can hold several types of carotenoids due to the similarity of their chemical structures [56]. Therefore, assuming that lutein can bond to all of these sites, the storage capacity of lutein on Lhcb protein is estimated at 4.1–8.1% in terms of mass ratio.

Most lutein/carotenoids involved in the photosynthetic and the nonphotochemical-quenching (NPQ) reaction are bound to Lhc protein-based antenna complexes. However, other photosynthetic proteins such as the core of photosynthetic systems and cytochrome b6f complex can bind carotenoids. Hence, the storage capacity of lutein throughout the photosynthetic system should be addressed.

Unfortunately, the amount of carotenoid associated with the entire photosynthetic system of algal species is not yet clear. Thus, the lutein storage capacity of the photosynthetic system can only be estimated. Since the core complexes of PSII and PSI are largely conserved from cyanobacteria to plants, the amount of carotenoids associated with PSII-LHCII complex in the model organism *Arabidopsis thaliana* was taken into account [57]. In *A. thaliana*, the basic unit of the PSII-LHCII complex is the protein complex C2S2M2. The protein mass of C2S2M2 is around 1.1MDa, and in this complex it binds 75 carotenoid molecules [58]. Therefore, assuming that the 75 carotenoids are all lutein, the storage capacity of lutein in the PSII-LHCII complex is around 3.9%. Since the antenna size and composition may vary in diverse organisms, the 4% of lutein storage capacity on this “structural form” is estimated based on the aforementioned knowledge.

### 2.2. Estimated Storage Capacity of Lutein in Lipid Droplets

It has been found that carotenoids could be massively concentrated in variety of lipid environments [59,60,61]. For example, over 50% (w/w) of β-carotene was stored in the specialized plastoglobules of *Dunaliella bardawil*. *Haematococcus pluvialis* was found to accumulate 40% of the dry cell weight (DCW) as cytoplasmic lipid droplets (CLD); 4% DCW of the astaxanthin was concentrated in the CLD. Similarly, the majority of lutein was also found concentrated in the plastoglobules of marigold petals [62]. Unfortunately, the mass ratio between lutein and the plastoglobules in the marigold is not yet apparent. Considering the effect that 20 wt % lutein could be sequestrated in corn oil and 10% astaxanthin has been stored in the CLD of *H. pluvialis* [63], it can be assumed hypothetically that lutein could have 10–20% storage capacity in the “oil form” because of the similarity in lutein and astaxanthin’s chemical structures.

### 2.3. Limits of Structural Lutein Content due to Its Function

The nature of lutein as a structural carotenoid determines that its contents will be low, as required to fulfill photochemical functions. The storage capacity of lutein in the structural form was estimated as 4% versus 10–20% in the oil form. To sequestrate 2% DCW of lutein in the structural form, the algal cell needs to produce 50% DCW of photosynthetic protein for lutein storage. Consider the optimum protein content of different algal strains: *Chlorella* spp. (50–60%, DCW), *Chlamydomonas* spp. (48%, DCW), *Scenedesmus obliquus* (50–56%, DCW) and *Dunaliella salina* (57%, DCW) [64]. It is almost impossible for algal strains to synthesize over 50% DCW of photosynthetic proteins for the 2% DCW of lutein sequestration as a structural pigment. The nature of lutein as a structural carotenoid determines that its content will not be very high in fulfilling photochemical functions in microalgae. The data used to calculate lutein storage capacity in LHCs is based on that of higher plants. However, the content of LHCs protein to total protein varies in different algal strains. Considering the conserved structure of LHCs in algae and higher plants, using the above method to estimate the lutein storage capacity in LHCs and lipid droplets is assumed acceptable.

Sequestering lutein in lipid offers a much greater potential, as microalgae could accumulate up to 40–60% DCW as lipids [65]. Based on the lutein storage capacity in the oil form, algal cells would only need to generate 10–20% DCW of lipids for the sequestration of 2% DCW of lutein. Therefore, lutein sequestration in “oil form” presents a significantly higher lutein storage upper limit than the “structural form.” Exploring the method to accumulate lutein in oil form could result in a higher lutein content in algal cells.

## 3. Limitation of Lutein Sequestration in Algal Oil Droplets

Theoretically, lutein sequestration in the lipid environment provides a higher capacity than bonding to the photosystems. However, lutein and lutein ester could be highly concentrated in the plastoglobule of marigold [52,66]. A massive amount of β-carotene was sequestrated in the special plastoglobules of *Dunaliella* spp. [41,67]. Astaxanthin and astaxanthin esters could accumulate in the cytoplasmatic lipid droplets (CLD) in *Haematococcus* spp. and *C. zofingiensis* [68,69,70]. Few studies have found that lutein can accumulate in the lipid droplet of microalgae species. Thus, several major barriers may prevent the sequestration of lutein in the lipid droplets algal species as discussed below.

### 3.1. Lutein Degradation without Proper Sequestration

Without proper sequestration, lutein is trapped on chloroplast membranes and increases the possibility of degradation under the stress of UV-light, heat, ROS and by the catalyzation of carotenoid cleavage dioxygenases (CCDs). The two hydroxyl-groups on the chemical structure of lutein trap it on the surface, or inside, the chloroplast’s inner membrane. A similar phenomenon has been investigated in vitro by exploring the effect of carotenoid incorporation into liposomes [71,72]. Lutein can be incorporated tightly with phospholipid membranes as its polar hydroxyl-groups intercalates into the membrane structure and interact with the polar sides and the membranes’ aqueous environment. The rigidity of the phospholipid membrane is positively correlated with the amount of lutein incorporated.

Therefore, without the assembly of lutein into photosynthetic proteins or the trafficking of lutein into the lipid droplet, lutein could be concentrated in the inner envelope membrane with the thylakoid membrane. This leads to feedback limitation on lutein biosynthesis and the degradation of lutein via photo-oxidation and carotenoid cleavage dioxygenase (CCDs) catalyzation [45,47].

Carotenoid cleavage dioxygenases (CCDs) constitute a widespread enzyme family consisting of an activation center on top of the structure and a Fe^2+^ ion, coordinated with four histidine residues [73,74]. The Fe^2+^ ion activates oxygen, which is involved with the reaction center. The activated oxygen cleaves the carbon-carbon double bond on the polyene chain of the carotenoid’s structure. The CCDs protein is located at the envelope and plastoglobuli membrane of arabidopsis [75,76,77]. It is also located on the chromoplast membrane of watermelon, tomato, carrot, cauliflower, papaya, pepper and citrus [78,79].

Although the effect of CCD expression on carotenoid accumulation was not evident in microalgae species, proteomics data of *C. reinhardtii* indicated that a protein located in eyespot was homologous to the CCDs of *Synechocystis* spp. [80]. Thus, CCDs may still degrade different kinds of carotenoids, including lutein, in microalgal species. Since lutein is vulnerable under ROS stress and easily degraded by CCDs without LHC storage, it is critical to better preserve lutein in this condition. The esterification of lutein could address this problem. However, lutein cannot be esterified in most algal strains, as discussed below.

### 3.2. Lack of Lutein Esterification Mechanism

Carotenoid esterification implies that one or two of the hydroxyl-group on the carotenoid is esterified with fatty acid. Carotenoid esters are common in flower petals such as marigold, senescent leaves, as well as fruits of potato plant, chili peppers, vegetables and some algal strains such as *Haematococcus* spp. and *C. zofingiensis* [28,68,81,82,83,84].

The esterification of hydroxyl-carotenoids has received extensive attention in recent years. The ester condition could provide an additional positive attribute that enhances several properties of hydroxyl-carotenoid. The ester condition significantly increases the stability of hydroxyl-carotenoid under UV stress, ROS stress and heat treatment [85,86,87,88].

Esterification of carotenoids may integrate carotenoids into different kinds of lipid droplets [82]. Studies suggested a relationship between carotenoid esters and lipid droplet development in plants [89]. For example, the xanthophylls could not be esterified in a tomato mutant, which was accompanied by decreased carotenoid accumulation and fewer fully developed plastoglobules in petals. Therefore, esterification could increase the stability of carotenoids both in vitro under UV light and heat treatments and could also integrate carotenoids into lipid droplets. Introducing the esterification reaction of lutein in microalgae could better preserve lutein under stress conditions. However, the access to lipid droplets remains another barrier for sequestration of lutein or lutein ester in microalgae.

### 3.3. Lipid Droplet Access Limitation

Three types of lipid droplets were found in microalgal strains: eyespot, βC-plastoglobule and cytoplasmic lipid droplets (CLD) [90,91,92]. The biogenesis of lutein, eyespot, and βC-plastoglobule occurs in the chloroplasts of algal cells. In theory, eyespot and βC-plastoglobule could be the sink for lutein sequestration. However, eyespot and βC-plastoglobule biogenesis are missing in most lutein-producing strains, and massive β-carotene accumulates only in the lipid vessels of a few microalgal species [67,92].

Most microalgae strains consist of CLD in the cytoplasm, which only accumulates an amount of neutral lipids [91]. However, the carotenoid-containing CLD has only been found in *Haematococcus* spp. and *C. zofingiensis* [68,69,83,93]. A β-carotene transport mechanism was suggested in *Haematococcus* spp., which channeled the β-carotene from the chloroplast to the CLD. β-carotene was then converted into astaxanthin and astaxanthin esters, which were sequestrated in the CLD of *Haematococcus* spp. [68]. For *C. zofingiensis*, the MVA pathway was triggered with the biogenesis of CLD, which synthesized astaxanthin and keto-lutein in the cytoplasm. Esterified astaxanthin and keto-lutein are concentrated in the CLD of *C. zofingiensis* [83]. Therefore, without biogenesis of lipid droplets in the chloroplast and the transport mechanism that channels lutein into the cytoplasm, it is difficult for lutein to access lipid droplets.

The accessibility of lutein to the lipid droplet must be “granted” in microalgal strains. Currently, the mechanism of the biogenesis of βC-plastoglobules in *Dunaliella* spp. is still unclear, as is the mechanism of the β-carotene trafficking through chloroplast membrane in *Haematococcus* spp. and *C. zofingiensis*. Exploring the two mechanisms depends on further proteomic research and a deeper understanding of the regulation of carotenoid accumulation in these microalgal strains.

## 4. Strategies to Overcome These Barriers

To overcome these barriers, systematic research on the mechanism of regulation of carotenoid sequestration in microalgae is warranted. Due to multiple barriers, enabling the sequestration of lutein in the lipid droplet may not be achieved by solving just one or two of these problems. However, investigating specific issues could create a foundation for addressing this problem. Therefore, the strategies are prioritized according to their feasibility with supporting discussions.

### 4.1. Lutein Esterification Gene and Mechanisms

The understanding of the carotenoid esterification mechanism has made significant progress in recent years. The Pale Yellow Patel 1 (*PYP1*) gene has been identified and isolated in a tomato mutant [89]. Evidence indicates that mutation of the *PYP1* gene results in the loss of xanthophyll esters. This is accompanied by decreasing total carotenoid yield and a reduction in fully developed plastoglobules in petals. Studies suggested that *PYP1* is highly relevant to the xanthophyll esterification reaction in tomato flowers [89]. This is further supported by a study characterizing the Pale Yellow Patel (*PYP*)/Xanthophyll esterase (*XES*) gene family in citrus [94]. Results show that the transcript level of *PYP1*, *2* and *6* are correlated with carotenoid ester content in citrus, and the expression of *PYP* accords with the low level of xanthophyll esters in yellow citrus. Similar results were found in petunias [95]. The *XES* from morning glory, tomato and marigold have been expressed in petunias. This results in a strong positive correlation between the expression of *XES* genes and xanthophyll esterification content. Besides, the expression of *XES* leads to an increase in the percentage of xanthophyll esters in transgenic mutants.

Unfortunately, the function of the *PYP*/*XES* gene has not yet been characterized in vitro. The enzymatic condition and the substrate preference are still unclear. It was found that xanthophylls were not esterified after the *XES* genes were expressed in petunia leaves. This implied that the *XES* might prefer a lipophilic environment, such as plastoglobules [95]. Therefore, comprehensive characterization of the *PYP*/*XES* function in vitro is needed before the heterologous expression of microalgae and other species.

Xanthophyll acyltransferase (XAT) was first isolated in bread wheat [82]. It was found that the function of this enzyme is necessary for carotenoid esterification, both in vitro and in vivo. The enzymatic condition of XAT was also characterized. Results show that XAT can catalyze the esterification reaction of lutein, zeaxanthin and β-cryptoxanthin with a wide range of acyl-donor substrates, such as triglycerides, 1,2-diglycerides, lysophosphotidylcholine, free fatty acids, 1,3-diglycerides and monoglycerides.

XAT can esterify lutein with a wide range of acyl-donor substrates. Triglycerides and free fatty acids can accumulate to high levels in the cytoplasm and the chloroplast of algal cells. Therefore, XAT is an excellent candidate to explore lutein esterification’s effect on the accumulation of lutein in microalgal cells.

### 4.2. Suppression of the Gene Expression of CCDs in Microalgae

Evidence from transgenic studies indicates that the expression of *CCDs* is critical to the regulation of carotenoid accumulation in plants. The high transcriptional level of *CCDs* results in white petals and low carotenoid content in lilies and chrysanthemums [96,97]. Similarly, a negative correlation between the expression of *CCDs* and carotenoid accumulation has been found in arabidopsis, peach fruit, and potatoes [98,99,100,101]. Furthermore, suppressing the expression of *CCDs* by RNAi interference results in a three to six times higher carotenoid content in the petal of the mutants than in the wild-type of chrysanthemum [97]. However, the color of the petals remained yellow in transgenesis lines, while the wild type lost its color. Conflicting results were found in morning glory and citrus [102,103]. The results indicated that the transcriptional level of *CCDs*, or the overexpression of *CCDs*, affected carotenoid accumulation.

In microalgae, the function and the localization of CCDs enzymes and the effect of *CCDs* expression on carotenoids accumulation are still poorly understood. Proteomics data of *C. reinhardtii* revealed that a protein located in the eyespot was homologous to the CCDs of *Synechocystis* spp. [80]. Several CCDs homologous proteins in other algal strains have also been found in the NCBI database, for example, *D. salina* (APW83743.1); *Scenedesmus* spp. (KAF8065651.1); *C. sorokiniana* (PRW57550.1). Therefore, investigating the correlation between *CCDs* expression and carotenoid accumulation could elucidate carotenoid accumulation in microalgae. Findings about the effects of *CCDs* expression on the carotenoid accumulation in plants suggest that suppressing the expression of this gene in green algae by genetic modification may increase carotenoid content in green algae.

### 4.3. Investigation of the Mechanisms of βC-Plastoglobuli Biogenesis in Dunaliella spp. and the Carotenoid Trafficking Mechanisms in Haematococcus spp. and C. zofingiensis

Investigating the mechanisms of biogenesis in βC-plastoglobules in *Dunaliella* spp. and of the trafficking of β-carotene through the chloroplast membrane in *Haematococcus* spp. and *C. zofingiensis* are systematic and complex studies. Exploring the two mechanisms requires additional proteomic research, further understanding of the regulation of carotenoid accumulation in these microalgal strains, and the advanced genetic toolbox in microalgal species.

### 4.4. Future Perspectives

The development of a cell factory for lutein production in microalgae demands further exploration on the regulation of lutein biosynthesis, degradation and sequestration in microalgal species. The regulation of lutein biosynthesis has garnered considerable attention in recent years. However, the sequestration of lutein plays a critical role in the accumulation of lutein. Future research on this topic is warranted.

Several critical barriers block lutein sequestration in lipid droplets in microalgal species. The exploration of lipid droplet biogenesis in the chloroplast, carotenoid cross membrane mechanisms, and carotenoid esterification mechanisms rely on an optimized genetic engineering toolbox for different microalgal species. Unfortunately, the toolbox is still in the formative stage for most microalgae.

Investigating similar mechanisms by using a model yeast strain such as *Saccharomyces cerevisiae* and *Yarrowia lipolytica*, with their relatively simple cell structure, and with a more mature genetic engineering toolbox, could help understand the carotenoid sequestration mechanisms.

The nonconventional oleaginous yeast *Y. lipolytica* has been engineered as a cell factory for production of different carotenoids due to several unique features [104,105,106]. On one hand, *Y. lipolytica* can accumulate a massive amount of acetyl-CoA as a precursor of the mevalonate pathway, which provides sufficient geranyl diphosphate (GPP), farnesyl diphosphate (FPP) and geranylgeranyl diphosphate (GGPP) as the intermediates for the accumulation of carotenoids [107,108]. On the other hand, large amounts of oil are accumulated in the lipid droplets of *Y. lipolytica* that provide a desired lipophilic container for carotenoid storage [104,107]. Although lutein biosynthesis has not been achieved in *Y. lipolytica*, due to the similarity of chemical structure its isomer zeaxanthin could be used to investigate carotenoid esterification mechanisms and other mechanisms of carotenoid sequestration.

## 5. Conclusions

Several microalgae species with high lutein content (~1% of the dry cell weight) have been reportedly isolated. However, compared with the level of β-carotene concentrates in *Dunaliella* spp. (up to 14% of dry weight) and astaxanthin in *Haematococcus* spp. (up to 5% of dry weight), several potential limiting factors for lutein accumulation in microalgae species must be identified and addressed to achieve higher levels for greater feasibility of commercialization. Since sequestration is critical for lutein accumulation, the concept of lutein storage capacity was proposed to estimate the lutein storage upper limit in lipid droplets and LHCs.

A lutein storage capacity of 4 and 10–20% was estimated in LHCs and lipid droplets, respectively. According to the optimum protein content in microalgalspecies, it is almost impossible to sequestrate 2% DCW of lutein in LHCs. Although enabling lutein to be stored in the lipid droplet could help increase lutein content in microalgae species, accessing lipid droplets and degradation in the inner envelope of the chloroplast are the main barriers to overcome to make it happen. Further exploration on lipid droplet biogenesis, carotenoid trafficking mechanisms, carotenoid esterification mechanisms and other carotenoid accumulation and regulation mechanisms may open future possibilities.

## Figures and Tables

**Table 1:** Lutein content and productivity of microalgae strains under desirable cultivation conditions.

Microalgal Strains	Lutein Content (mg/g)	Lutein Productivity (mg/L/d)	References
*C. sorokiniana* FZU60	9.57	11.57	[20]
*Parachlorella* sp. JD-076	11.8	25	[22]
*Desmodesmus* sp. F51	5.56	5.22	[23]
*Scenedesmus obliquus* FSP-3	4.52	4.15	[17]
*C. sorokiniana* MB-1	5.86	2.39	[24]

**Table 2:** Examples of mutagenesis and metabolic engineering strategies for altering lutein content in microalgae strains.

Microalgal Strains	Strategy	Lutein Yield (mg/g)	References
*C. sorokiniana* MB-1-M12	Random mutagenesis	7.52	[24]
*C. sorokiniana* mutant DMR-5&8	Random mutagenesis	7.0	[16]
*C.zofingiensis* mutant CZ-bkt1	Dysfunction of *BKT1* by chemical mutation	13.81	[28]
*Chlamydomonas reinhardtii*	Overexpressing *PSY* gene from *Dunaliella salina*	2.2-fold increase of lutein content	[29]
*C. reinhardtii*	Overexpressing *PSY* gene from *C. zofingiensis*	2.2-fold increase of lutein content	[30]
*C. zofingiensis*	Overexpressing *PDS* gene	Total carotenoid content increased by 32.1%	[31]
*C. reinhardtii*	Overexpressing *OR* gene from *A. thaliana*	1.9-fold increase of lutein content	[32]
